# Brewer’s Spent Grain, Coffee Grounds, Burdock, and Willow–Four Examples of Biowaste and Biomass Valorization through Advanced Green Extraction Technologies

**DOI:** 10.3390/foods12061295

**Published:** 2023-03-18

**Authors:** Massimiliano Errico, Jose A. P. Coelho, Roumiana P. Stateva, Knud V. Christensen, Rime Bahij, Stefania Tronci

**Affiliations:** 1 Faculty of Engineering, Department of Green Technology, University of Southern Denmark, 5230 Odense M, Denmark; 2 Instituto Superior de Engenharia de Lisboa, Instituto Politécnico de Lisboa, Rua Conselheiro Emídio Navarro 1, 1959-007 Lisboa, Portugal; 3 Centro de Química Estrutural, Institute of Molecular Sciences, Instituto Superior Técnico, Universidade de Lisboa, Av. Rovisco Pais, 1049-001 Lisboa, Portugal; 4 Institute of Chemical Engineering, Bulgarian Academy of Science, 1113 Sofia, Bulgaria; 5 Dipartimento di Ingegneria Meccanica, Chimica e dei Materiali, Università degli Studi di Cagliari, 09123 Cagliari, Italy

**Keywords:** biomass, biowaste, BSG, burdock, spent coffee grounds, willow

## Abstract

This paper explores the transformation of biowastes from food industry and agriculture into high-value products through four examples. The objective is to provide insight into the principles of green transition and a circular economy. The first two case studies focus on the waste generated from the production of widely consumed food items, such as beer and coffee, while the other two examine the potential of underutilized plants, such as burdock and willow, as sources of valuable compounds. Phenolic compounds are the main target in the case of brewer’s spent grain, with *p*-coumaric acid and ferulic acid being the most common. Lipids are a possible target in the case of spent coffee grounds with palmitic (C16:0) and linoleic (C18:2) acid being the major fatty acids among those recovered. In the case of burdock, different targets are reported based on which part of the plant is used. Extracts rich in linoleic and oleic acids are expected from the seeds, while the roots extracts are rich in sugars, phenolic acids such as chlorogenic, caffeic, *o*-coumaric, syringic, cinnamic, gentisitic, etc. acids, and, interestingly, the high-value compound epicatechin gallate. Willow is well known for being rich in salicin, but picein, (+)-catechin, triandrin, glucose, and fructose are also obtained from the extracts. The study thoroughly analyzes different extraction methods, with a particular emphasis on cutting-edge green technologies. The goal is to promote the sustainable utilization of biowaste and support the green transition to a more environmentally conscious economy.

## 1. Introduction

Process optimization is a fundamental tool within process system engineering. Following the definition given by Seider et al. [1], process optimization is an underspecified problem where the number of variables is higher than the number of equations. It results in the selection of a set of decision variables to be iteratively adjusted to achieve a solution that satisfies a specified objective. In the past, the most common objective was related solely to the economy of the process considered. During the 1970s, at the time of the energy crisis, the priority was the minimization of energy consumption, allowing big consumers, such as refinery plants, to stay competitive in the market. In this context, Pinch Technology was developed to design heat exchanger networks to minimize the use of external utilities through the integration of process streams [2,3]. The methodology was applied to different processes with energy savings of up to 20–40% [4]. In the late 1980s, the United Nations Commission on Environment and Development released the Brundtland Report, a call to embrace a sustainable model of development that addresses the needs of the present without compromising the ability of future generations to meet their own needs. The report was a turning point in recognizing the importance of the environmental impact of a specific production and its possible implementation in the design techniques available. 

Moving forward, the European Green Deal is driving research and society toward the full implementation of those sustainable principles introduced over 30 years ago. The European Green Deal has the objective to reach a zero net emission of greenhouse gases by 2050 and to decouple the economic growth from non-renewable resources [5]. This type of commitment requires a profound rethinking of the production routes for most of the chemicals produced from non-renewable sources or through synthesis reactions of oil-derived intermediates. The new approach to industrial organization follows the circular economy principles powered by the “Cradle to Cradle” perspective. While the linear economy approach is “make, use, dispose”, a circular economy moves to the 3R approach (reduce, reuse, recycle), which can open up new avenues and opportunities. To realize the ambitious goals of the circular economy, innovative and advanced, predominantly mild, essentially waste-free techniques, processes, and technologies are required. 

This change requires not only a change in feedstock type, but also a new and efficient way of processing new sustainable matrices. In other words, is the interest in unit operations that are consolidated in the present processing industry expected to fade? Together with the green transition, is there also a transition to different unit operations? Should a different process layout, with respect to the classical reactor plus separation system presented in most of the chemical process design books, be expected? 

To provide clear-cut answers to those questions would be pretentious. However, it can be assured that extraction processes, in particular the recovery of valuable compounds from solid matrices, earn a certain spot among the different unit operations used to fully exploit various biomasses. In general, techniques that will allow the recovery of high value-added products with a wide spectrum of applications from renewable sources without damaging one or more of the extracts, particularly those that are heat sensitive, will be the key to the green transition.

In this review, four examples are considered that cover different types of biomass. The first discusses brewer’s spent grain, a waste produced in high quantity and largely available. The second presents spent coffee grounds (SCGs) that are also produced in large quantities and can be collected from households, restaurants, and cafeterias. The third example outlines the prospects for the valorization of a renewable, but highly underused biomass, *Arctium lappa*—more commonly known as greater burdock or simply burdock. The last case explores the potential of willow as a source for extracting components for the pharmacological industry. Despite the different varieties of willow, here, the fast-growing shrubs commonly used either for protecting fragile areas from soil erosion or as raw material for rural handcrafts are considered.

Altogether, this set of biomasses represents different cases where the biomasses are generated as wastes from the production of beverages consumed worldwide or cultivated without competing with food-related crops. Notwithstanding this diversity, for all of them, it is possible to define a sustainable way to recover valuable compounds based on green extraction methods.

## 2. Brewer´s Spent Grain

According to BarthHass, one of the world’s leading suppliers of hop products and hop-related services, the global beer production in 2021 was approximately 1.86 billion hL (hectoliters) [6]. Of this amount, 33.6% was produced in the Americas, 30.7% in China, 28.0% in Europe, and 7.7% in Africa. The popularity of this beverage all over the world is evident, and even if there are different beer types and different traditions associated with its production, there is a general similarity in the production lines and, consequently, the generation of waste streams. 

Beer production follows different steps in which malted cereal grains are treated to produce a wort to be fermented by yeast, converting sugars into ethanol, carbon dioxide, and other metabolites that contribute to the product flavor. In particular, BSG is separated in the lautering step in order to obtain a clear wort for the fermentation step. A deeper analysis of beer production can be found in Briggs et al. [7]. The proportion between feed and BSG is 100–130 kg of fresh BSG produced for each 100 kg of malt [8]. This also corresponds to about 20 kg of wet BSG for each hectoliter of beer produced [9]. Considering the figures given about beer production, BSG emerges as a biowaste available in large amounts whose valorization could benefit breweries’ economy and the sustainability of the overall production.

It is worth considering how the approach to sustainability and the general concept of biocascades is relevant now more than ever. One of the world’s leading social investment networks, eToro, estimated that the price of producing one pint of beer has risen by 62% in the last two years [10]. The evaluation was based on the fundamental commodities used in the beer production process, namely, the feedstock, the aluminum for the cans, the fuel for the process and transportation. Nevertheless, on the consumer side in the UK since 2020, the price increase of one pint of beer was approximately 7% [11], suggesting that brewers are covering part of the cost. In this scenario, complicated further by the war in Ukraine, the possibility of compensating for the reduced profit margin by the valorization of the waste streams is a fundamental option to be explored. 

At the present time, 70% of the BSG produced is used as animals feed, 10% for biogas production, and 30% is disposed of as landfill [8]. Its use as animal feed is an easy way to redirect this waste and improve the economy of livestock feeders, as discussed by *The Denver Post* [12]. Considering the monetary value associated with dry BSG for animal feed, this was quantified at EUR 13.95/ton (metric ton), while if used as a natural source of tocopherols for the isolation of nutraceuticals, the value increases by an order of magnitude reaching EUR 300/ton [13]. 

In order to identify possible pathways for a full BSG valorization, in the next paragraph, its composition is discussed together with some value-creating processes proposed in the literature.

### 2.1. BSG Chemical Composition

Depending on the brewery conditions used, barley variety, and harvest time, different BSG compositions are expected. A comparison of different BSG characterizations were reported by Lynch et al. [9], Lisci et al. [14], Mussatto [15], and Milew et al. [16], among others. 

Table 1 summarizes the BSG composition reported by a selection of different authors based on the main components or class of components.

From Table 1, it is noticed that the BSG characterization is generally reported in a heterogeneous way, making the comparison between different studies difficult. The difficulty arises not only from the differences in the nature of the substrate, but also from the analytical technique used for the quantification of the different fractions. 

A representative characterization was reported by Agrawal et al. [22], where the BSG average composition is: cellulose (15–25%), hemicellulose (20–40%), lignin (10–20%), starch (1–10%), lipids (3–10%), proteins (15–30%), minerals (<0.5%), phenolics (1–2%), and vitamins (<0.5%). 

According to Mussatto [15], Meneses et al. [23], and Krausova [24], the mineral fraction is mainly composed of silicium, phosphorous, calcium, magnesium, and sulfur, with other components such as iron, copper, and zinc in minor amounts. 

Extractives were identified by Mussatto and Roberto [17] as waxes, fats, gums, starches, resins, tannins, and essential oils. Meneses et al. [23] quantified, by difference, the amount of extractives to 10.73 ± 0.32 g per 100 g of dry matter, focusing their analysis on the recovery of phenolic compounds due to the recognized antioxidant properties. 

The protein fraction represents a natural source of different food ingredients. Hordeins (A, B, and C) are the main fraction of proteins, followed by glutenins, and albumins as the less abundant portion [25]. 

Niemi et al. [26] recovered a fraction of lipids equal to 11% of the dry material, which is aligned with most of the values reported in Table 1. Of this fraction, 55% were triglycerides, 30% were free fatty acids, and a minor part was phospholipids and diglycerides accounting for 9.1% and 5.7%, respectively. The most abundant lipids were linoleic, palmitic, and oleic acids. A detailed characterization of the lipidic fraction is reported by del Rio et al. [27].

### 2.2. BSG Valorization Technologies

Solid–liquid extraction represents the simplest way to recover different components based on their affinity for the solvent chosen as the extractant. 

Bonifacio-Lopez et al. [28] used absolute ethanol, water, an aqueous solution of 80% *v*/*v* of ethanol, and an aqueous solution of 60% *v*/*v* of ethanol in order to recover antioxidant-rich extracts. The 60% ethanol solution resulted in obtaining the best extract in terms of phenolic compounds, with catechin and vanillic acid producing the most concentrated ones. The total phenolic compounds extracted were quantified in 604.6 μg per g of BSG in the case of the 60% ethanol solution, while only 43.2 were obtained in the case of absolute ethanol. Besides the extraction yield in terms of the components extracted, the same authors completed the analysis reporting the cytotoxicity. The result of this analysis was that 80% ethanolic extracts were considered safe up to 10 mg mL^−1^ while 60% extracts are only up to 1 mg mL^−1^. Zuorro et al. [29] tested different concentrations of acetone–water and ethanol–water solutions, showing that the solution with a 60% *v*/*v* of organic solvent solution achieved the highest extraction yield and the highest antioxidant activity. 

Ancuta Socaci et al. [30] tested 12 solvents including water, hexane, ethyl acetate, acetone, methanol, ethanol, and different concentrations of aqueous solutions of water–methanol, water–ethanol, and water–acetone. The results showed that 60% *v/v* solutions of ethanol and 60% *v*/*v* solutions of acetone performed better regarding the total phenolics and the total flavonoids extracted. Interestingly, the authors observed that the extracts with the lowest phenolic content are associated with high antimicrobial activity against different strains of Gram-positive and Gram-negative bacteria (*Staphylococcus aureus*, *Pseudomonas aeruginosa*, *Listeria monocytogenes*, *Salmonella typhimurium*, *Escherichia coli*, *Candida albicans*). The result, which seems surprising, could be associated with the fact that solvents such as hexane or ethyl acetate are not the most efficient in extracting antioxidant compounds, but they may have an affinity for compounds with relevant antimicrobial properties. 

Together with conventional solid–liquid extraction techniques, enhanced methods have been introduced to reach higher yields and to reduce the extraction time and the amount of solvent used. Moreira and Morais [31] used microwave-assisted extraction (MAE) for the recovery of polyphenols using a 0.75% solution of NaOH as the solvent. At the optimal extraction conditions of 100 °C, 15 min extraction time, and 20 mL of solvent per g of dried BSG, the yield of ferulic acid was five times higher than the conventional alkaline hydrolysis extraction technique. Alonso-Riano et al. [32] compared mechanical stirring and ultrasound-assisted extraction in terms of total polyphenol content and extraction kinetics using water as the solvent. Comparing the two methods, for the MAE, the extraction was completed after 20–30 min, while 24 h were necessary for the mechanical stirring. Together with the reduction in the extraction time, the authors also reported a 30% increase in the extraction yield when ground BSG was considered. However, the amount of ferulic acid recovered through the MAE resulted in significatively lower yields than using acid, alkaline, and enzymatic hydrolysis-based methods. 

An alternative to conventional and enhanced solid–liquid extraction techniques is supercritical fluid extraction (SFE) belonging to the green process concept [33]. In fact, SFE is considered a green and innovative technique that, in comparison to conventional approaches, operates with a reduced processing time, consumes less solvent and energy, and diminishes the carbon footprint [34]. Fernandez et al. [13] proposed a two-step process where the BSG is pretreated by vacuum-drying to reduce the initial moisture content from 78% to 58% *w*/*w* and then used for SFE targeted to tocopherol recovery. The authors optimized the SFE step in the operating parameter range of 10.35 MPa and 313–353 K for raw and milled BSG. As expected, better results were reported for the milled BSG, with the highest yield measured at the lowest temperature and the highest pressure. Under those conditions, the concentration of tocopherol in the extract was 2 ppm. Spinelli et al. [35] extended the optimization of the operating conditions by studying the extraction performance in the temperature range of 313–333 K and pressure of 15–35 MPa, using only CO_2_ and CO_2_ with 20, 40, and 60% *v/v* of ethanol. The extracts were compared based on the extraction yield and their total polyphenolics, flavonoids, and antioxidant activity. The results reported by the authors highlighted how the co-solvent was the variable that mainly influenced the extraction performances. The best results in terms of total phenolic and flavonoid compounds together with good antioxidant properties were reported for a pressure of 35 MPa, a temperature of 313 K, and 60 % *v/v* ethanol. It should be noted that the best concentration of the co-solvent also corresponds to the most common optimal conditions reported for the conventional solid-to-liquid extraction previously discussed. 

Other articles studied the optimization of the SFE conditions for BSG using different compounds as the target of the extraction efficiency. Among these, Ferrentino et al. [36] and Alonso-Riano et al. [37] completed the analysis, reporting the fatty acid profile of the extract. The main fatty acid present was linoleic acid and its relative amount was not influenced by the use of ethanol as a co-solvent. 

## 3. Spent Coffee Grounds 

### 3.1. Importance of Spent Coffee Grounds

According to the ICO, coffee consumption in 2020/21 was 9.98 × 10^6^ metric ton, with Europe being one of the biggest consumers with 32.5% of the total amount, followed by Asia and Oceania with 21.9% [38]. It is estimated that for each kilogram of coffee consumed, around 0.91 kg of waste is produced, so it is important to look for added value and innovative solutions for the valorization of the huge quantities of spent coffee grounds (SCGs) produced. These solutions are motivated by solving the environmental problems contributing to implement the circular economy approach, targeted at a “zero waste economy” in which waste is used as a raw material for new goods and applications.

Besides lignin, cellulose, hemicellulose, and other polysaccharides [39], the recovery of the lipid fraction is of primary interest for this type of raw material. Compounds such as triacylglycerols (TAGs), diacylglycerols (1,2 DAGs), diterpene alcohol esters, fatty acids, sterols, caffeine cafestol, and kahweol [40,41,42,43] have an impact on human health and have been studied regarding their relation to anticarcinogenic [43] and antioxidant effects [44,45].

As discussed extensively in the literature, SCGs are a rich source of value-added products such as antioxidant compounds or functional additives [46,47]. Their importance as a source of energy has also been analyzed by several authors [48,49], either through the transformation of triacylglycerols to fatty acids and consequently to their esters to produce biodiesel, or as the final residue, which, after of several extraction methods, can be burned for energy production.

Supercritical fluid extraction (SFE), in the form of supercritical carbon dioxide (scCO_2_), has some advantages over traditional solid–liquid extraction techniques. As it is a flexible process due to the possibility of the continuous modification of the solvent power/selectivity of the supercritical fluid, allows the elimination of polluting and hazardous organic solvents, and it is easy to remove solvents from the extracts, SFE has been applied to lipid extraction from SCGs [50,51,52].

### 3.2. Extraction of Lipids from SCGs with scCO_2_

The extraction of the lipid fraction from SCGs is performed employing extraction with scCO_2_, either neat or with the addition of co-solvent(s) such as ethanol, isopropanol, and ethyl lactate. The results obtained were compared with the conventional Soxhlet on the basis of yield and composition of the extracts recovered. Moreover, the influence of the operating conditions was also examined.

Coelho et al. [50] performed SCG extraction with pure scCO_2_ at two temperatures (313 and 333 K) and three pressures (20, 30, and 400 MPa) using a flow rate of 1.9 × 10^−3^ kg·min^−1^. Moreover, in the extractions applying CO_2_ with different % mol fractions of co-solvents, specifically ethanol (sCO_2_ + EtOH, 5 and 10%), isopropanol (sCO_2_ + iPrOH, 10%), and ethyl lactate (sCO_2_ + EL, 10%), the final value of the CO_2_ flow rate was adjusted to be the same as that with pure CO_2_.

Coelho et al. [50] examined the combined influence of temperature and pressure, and the type of co-solvent used. Thus, it was demonstrated that when co-solvents are added, it is possible to increase the yield up to 12.3%. For example, when 10% of EtOH is used, the increase in the pressure from 20 to 30 MPa at *T* = 333 K not only enhanced the yield, but also considerably lowered the extraction time. However, for the other two co-solvents (iPrOH and EL), and at the lower temperatures applied, increasing the pressure did not influence the yield to any considerable extent. Nevertheless, when 5% EL was used, regardless of the temperature and pressure applied, a yield commensurable with that obtained with 10% ethanol and at the highest temperature and pressure applied (333 K and 30 MPa) was realized. 

Except for the lower pressure conditions of 20 MPa, 313 K, and 5% ethanol, where similar yields to hexane are obtained, there is a decrease in the extraction time of 50–70% when the more environmentally friendly supercritical fluid technique is used. These results with scCO_2_ agree with those of other authors [52,53,54].

### 3.3. Influence of Operating Conditions on Lipidic Composition of SCG Extracts

In addition to studying the influence of operating conditions on the yield, their impact on the composition and quality of the oils recovered was examined. The information obtained is of primary importance and can be used to guide the investigators on which technique and/or pressure, temperature, and co-solvents should be applied to achieve oils with a certain composition and variety of potential applications.

**Figure 1:**
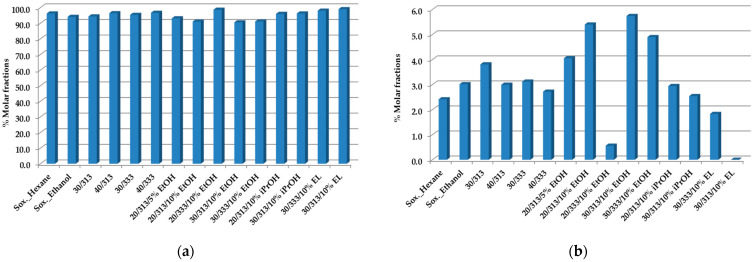
TAGs (**a**) and 1,2 DAGs (**b**) composition of spent coffee oils obtained by Soxhlet with hexane, ethanol, and scCO_2_ extraction at a flow rate of F = 1.8 × 10^−3^ kg·min^−1^, as established by ^1^H-NMR quantitative analysis [50]. All values represent % molar fractions.

Figure 1a,b shows the total amount of triacylglycerols (TAGs) and diacylglycerols (1, 2 DAGs), respectively, obtained from the SCGs.

The changes in the composition of the TAGs with the extraction conditions and methods are relatively lower, also due to their higher values in the samples, the main constituents of which are the oils. These results are in perfect agreement with others in the literature [51]. The total composition of the esters of fatty acids, analyzed by GC-Fid, was compared with the results of the TAGs by ^1^H-NMR, showing very good agreement [50].

With regard to the DAGs, and particularly the case at 20 MPa, 333 K, and scCO_2_ + 10% ethanol, a lower value was achieved. The maximum % molar fraction was obtained with ethanol as the co-solvent under the other extraction conditions. 

**Figure 2:**
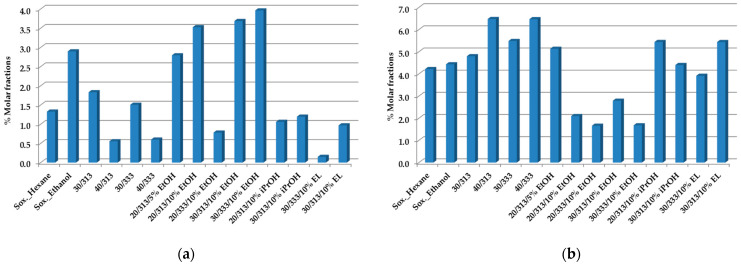
Caffeine (**a**) and cafestol (**b**) composition of spent coffee oils obtained by Soxhlet with hexane, ethanol, and scCO_2_ extraction at a flow rate of F = 1.8 × 10^−3^ kg·min^−1^, as established by ^1^H-NMR quantitative analysis [50]. All values represent % molar fractions.

Figure 2a,b shows the total amount of caffeine and cafestol, respectively.

The variations of these two compounds in the oils obtained allow us to substantiate that the presence of ethanol as a co-solvent increases the amount of caffeine extracted and decreases the content in cafestol. These variations may be associated with the increase in the polarity of the scCO_2_ with the addition of ethanol and consequent interactions with these two compounds.

**Figure 3:**
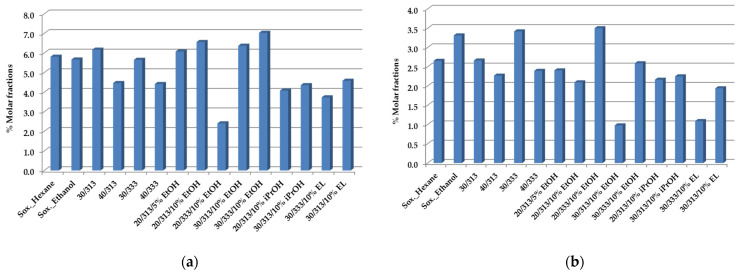
16-O-Mecafestol (**a**) and kahweol (**b**) composition of spent coffee oils obtained by Soxhlet with hexane, ethanol, and scCO_2_ extraction at a flow rate of F = 1.8 × 10^−3^ kg·min^−1^, as established by ^1^H-NMR quantitative analysis [50].

Finally, two more important compounds are shown in Figure 3a,b: 16-O-Mecafestol and kahweol.

The analysis of the influence of the main extraction conditions, namely, the temperature, pressure, and type of co-solvent applied on the oil yield, and the profile of the principle components outlined the possible paths to follow for the transformation of SCGs to value-added lipid constituents. 

Thus, the oil yield is increased favorably by raising the pressure and temperature. In addition, the use of a co-solvent allows a 20% increase in the final oil yield. Moreover, the increase in the polarity of scCO_2_ with the addition of co-solvents such as ethanol allows a growth in the % of molar fraction of the polar compounds in the oils, as is the case for caffeine (Figure 2a).

The results clearly demonstrate the potential of SCE as a green advanced technology for extracting oils from SCGs, leading to a reduction in the extraction time and the possibility of changing the composition of the extract through the adjustment of the best scCO_2_ conditions and use of the co-solvent.

## 4. Burdock

**Figure 4:**
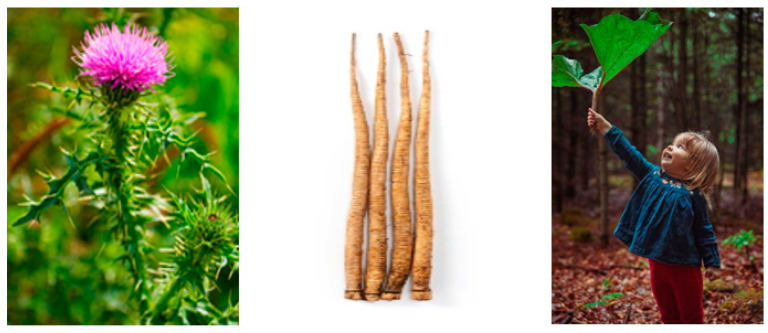
*Arctium lappa* plant, roots, and leaves.

*Arctium lappa* is the largest plant of the Asteraceae family. The plant is native to Eurasia, where it has been growing wild since ancient times. Burdock was brought to North America by European settlers, and due to its unpretentious nature, has spread easily. Consequently, in some countries, it is considered a weed and/or an invasive species. The plant’s leaves are over 1 m in diameter, and the stem reaches up to 3 m high (Figure 4). The major root (with few branches) is 3–6 cm in diameter, with a weight of about 450 g and can extend almost one meter in length (Figure 4). 

*Arctium lappa* is known by many names in different countries—edible burdock, beggar’s buttons, niúbàng in China, gobo in Japan, bardana in Brazil, repei in Bulgaria, etc. It has been extensively cultivated for over 3000 years in Asia and has been used as a nutritious vegetable in a variety of dishes. For centuries, burdock has been an important part of traditional Chinese medicine—e.g., root–water infusions have been widely applied as a diuretic, a laxative, and an antiseptic as well as to treat skin problems and external wounds and to increase male potency [55]. In medieval Europe, burdock roots were also used as a vegetable, but its popularity has since declined substantially. At present, the leaves and young shoots are consumed as a vegetable, while in Great Britain, a popular carbonated soft drink named “dandelion and burdock” has burdock roots as one of its two main ingredients. 

### 4.1. Burdock’s Physiological and Pharmacological Effects

Over the years, it has been shown that the anti-inflammatory effects of burdock are due to the presence of powerful antioxidants. Consequently, the attention of investigators has focused on examining the biochemical composition of the plant. For example, Liu et al. [56] commented that owing to the richness of secondary metabolites, both the seeds and roots of *A. lappa* possess a wide spectrum of physiological effects, namely, in vitro antioxidant and free radical scavenging activities, and anti-inflammatory effects, while the lignans present in the seeds exhibit a variety of biological properties such as in vitro and in vivo anticancer, antioxidant, antibacterial, antiviral, anti-inflammatory, and immunosuppressive activities. 

In the recent comprehensive review conducted by Yosri et al. [57], the pharmacological effects of burdock were extensively discussed and presented. It was reported that numerous preclinical studies (in vitro and in vivo) demonstrated that burdock exhibits a superfluity of biological activities, among which can be mentioned anti-cancer, anti-tumor, anti-diabetic, anti-viral, anti-obesity, anti-constipation, immune- and neuroprotective, and angiogenic activities, as well as ameliorating cerebral ischemia. 

Yet, despite the many proven positive effects on human health and wellbeing, the burdock plant biomass remains largely underused and, to a certain extent, even neglected. Moreover, there is a limited number of articles published in the open literature devoted to the valorization of this abundant sustainable and renewable source. 

### 4.2. Burdock Biomass Transformation into High-Value Bioactives by Innovative Techniques

As known, the common way for transforming plant matrices to high-value bioactive secondary metabolites is through the use of extraction techniques that apply either organic solvents (e.g., Soxhlet, two-phase solvent extraction), ultrasonication [58], or ionic liquid-based simultaneous ultrasonic and microwave-assisted extraction [59].

For example, Aboutable et al. [60] identified bioactive lignans and phenolics in extracts recovered by sonication at ambient temperature with 70% methanol from the roots, leaves, and seeds of *A. lappa* grown in Egypt. It was demonstrated that, e.g., genistein and chlorogenic acid were present both in the seeds and roots, cynarin and rutin were found in the roots, and lappaol A and F were only found in the seeds. 

Liu at al. [56] argued that although previous investigations only confirmed the presence of caffeoylquinic acids in burdock roots while lignans were found in the seeds and leaves, little was known about caffeoylquinic acids in the plant’s seeds, and lignans in its roots. They performed, for the first time, extensive qualitative and quantitative analyses of caffeoylquinic acids and lignans in the methanolic crude extracts of both the roots and seeds of six various burdock genotypes originating from different provinces in China. The authors’ conclusion was that the significant variations in the composition of hydroxycinnamic acids and lignans in the seed and root extracts are a consequence of the different burdock genotypes grown in different Chinese provinces.

Information about the fatty acids and esters detected in methanolic, *n*-hexane extracts of *A. lappa* was summarized in a recent review from Wang et al. [61]. Some of the identified compounds were oleic, palmitic, linoleic, linolenic, and stearic acids, methyl palmitate, and methyl stearate. Golbaz et al. [62] recovered extracts by hydrodistillation from burdock roots and reported that the main class of compounds found were sesquiterpenes (41.4%), phenyl propane (7.5%), and aliphatic components (4.8%).

Nowadays, however, the world’s focus has shifted from using large amounts of solvents for industrial-scale extractions to more selective extraction techniques using less environmentally harmful methods that operate under mild conditions. These methods can provide numerous advantages and can significantly reduce the drawbacks of organic solvent extractions, among which are the application of high temperatures and long extraction times, which might cause thermal degradation of the bioactive compounds. 

Despite the obvious benefits, efforts have remained focused mainly on the recovery of burdock extracts using organic solvent extractions. This literature review revealed that with regard to the recovery of secondary metabolites from *A. lappa*:

- The techniques and operating conditions applied for the recovery of bioactives might influence the quality of the extract.
- The genotype, seasonality, and cultivation conditions of the burdock plant in different countries can directly influence their chemical composition and metabolite content, as changes in the conditions of the place where the plant grows can significantly alter its capacity for metabolic homeostasis [63,64,65].

In what follows, the focus will be on the application of alternatives to organic solvent extractions for the transformation of burdock biomass to high value-added substances. These methods apply compressed fluids and liquids under pressure and at elevated temperature (pressurized liquid extraction, PLE). The first includes compressed propane and supercritical CO_2_, either neat or with Generally Regarded as Safe (GRAS) co-solvents such as ethanol. The PLE examined is either single or multistep [66].

Rodriguez et al. [67] and Stefanov et al. [66] examined the application of the above innovative green techniques for the recovery of bioactives from burdock roots, while Custódio de Souza et al. [68,69] examined the same from the plant leaves. Recently, Stefanov et al. [66] were the first to report the use of these advanced methods for the recovery of oil from burdock seeds. 

The extracts were evaluated by yield, total phenolic content, and antioxidant activity, and then qualified and quantified by a number of analytical methods (GC-FID, GC-MS, LS-MS/MS). Additionally, the yield and composition of the oils and extracts were compared with those obtained by Soxhlet extraction with different solvents (*n*-hexane, ethanol, methanol, ethyl acetate, etc.).

### 4.3. Influence of Techniques and Operating Conditions on Extracts’ Composition

Stefanov et al. [66] analyzed the composition of selected *A. lappa* seed oils recovered using different techniques. It was demonstrated that they contained saturated and mono-, di-, and polyunsaturated (MUFA, DUFA, PUFA) acids. The acid identified in the largest quantity was the essential linoleic acid at 61.2%, regardless of the method (either scCO_2_ + ethanol, propane, or Soxhlet + *n*-hexane) applied to obtain the respective oils. With a reference to linoleic acid, burdock seed oil was identified as the fifth richest among the vegetable oils, being inferior only to safflower, grape, and Silybum marianum oils, and commensurable with sunflower oils. Linoleic acid, a representative of omega-6 polyunsaturated fatty acids, is the most consumed essential fatty acid in the human diet, and has a wide range of applications in cells—from forming parts of the cellular membranes to being the source for many bioactive molecules through enzyme oxidation, elongation, or desaturation. Linoleic acid also has a wide range of clinical uses. The quantities of the acid in the roots, though lower (46–48%), still dominate the roots’ fatty acids and places the burdock root extracts among the top seven. 

Oleic acid was the second most abundant in the seeds oil, with quantities around 28%. Stearic acid was also detected with amounts around 3.6%. For both oleic and stearic acids, the amounts were not influenced by the method employed. Stearic acid is reported to have health benefits, e.g., it lowers LDL (“bad”) cholesterol and is neutral with respect to HDL (“good”) cholesterol, and hence is an important alternative to the currently widely used trans or other saturated and unsaturated fatty acids in the food industry.

Furthermore, it should be taken into consideration that burdock seed oils have a high index of unsaturation (about 1.5 vs. about 1 for spent coffee ground oil). Hence, the oils can be used as an appropriate source of unsaturated fatty acids suitable for the cosmetic and food supplement industries.

The amounts of fatty acids in the roots differ from those identified in the seeds. Thus, the quantity of saturated palmitic acid in the roots is around 31% regardless of the extraction method applied, which is almost six times higher than the quantity registered in the seeds. On the other hand, the quantities of the DUFA linoleic acid are lower (in the range 46–50%) and are influenced by the method applied. The highest amount of the acid is detected in the extract recovered by PLE with methanol (50.2%), followed by PLE with methanol/water, which is commensurable with that of Soxhlet methanol (48.3, 48.4%, respectively). 

The quantities of stearic acid in the root extracts vary from 1.2% (PLE with methanol) to 4.6% (second step of the three-step consecutive scCO_2_ extraction + methanol/water). 

With regard to the burdock roots, firstly, it should be noted that a high content of sugars (not detected in the seed oils), namely, fructose and sucrose, were registered. However, the quantities depended on the extraction method applied. The highest amounts were achieved when three-step consecutive scCO_2_ extraction with methanol/water as a co-solvent was applied, e.g., fructose at 67.97 and sucrose at 13.80 expressed as a relative percentage of all compounds identified. 

LC-MS/MS analyses detected some phenolic acids (both hydroxycinnamic and caffeoylquinic acid derivatives, and hydroxybenzoic acids derivatives) as well as several subgroups of the complex secondary metabolites belonging to the flavonoids family both in the seed oils and root extracts, among those being representatives of the flavonols, flavones, flavan-3-ols, flavanones (e.g., hisperidin, naringenin), and proanthocyanidins (e.g., procyanidin B1 and B3) subgroups.

The root extracts are very rich in phenolic acids—outstanding high quantities of chlorogenic as well as caffeic, gentisic, *o*-coumaric, syringic acids, to name just a few, were identified and quantified. 

As known, chlorogenic acid has proven antioxidant activities and exhibits a wide spectrum of biological effects, such as antimutagenic, antiviral, anticarcinogenic, antidia-betic, DNA protective, and neuroprotective effects. Moreover, chlorogenic acid also shows inhibitory activity against the hepatitis B virus (HBV) in vivo and in vitro.

Thus, if the target compound for the root biomass transformation is chlorogenic acid, then the following should be taken into consideration: the highest quantity of the acid (3652.913 ng/mg) is identified in the root extract recovered by three-step consecutive scCO_2_ extraction with methanol/water at *T* = 313.15 K and *p* = 20 MPa, while the lowest (1625.742 ng/mg) is detected in the extract obtained by the same technique, but at the higher temperature of 353.15 K. 

In contrast, the quantities of phenolic acid in the seed oils are much lower. Thus, the amounts of chlorogenic acid are over 100 times lower, and strongly depend on the method of recovery. The highest amount (around 32.3 ng/mg) is identified in the oil obtained by scCO_2_ + ethanol at *T* = 313.15 K and *p* = 20 MPa. 

However, another path for the root biomass transformation is to obtain an extract rich in caffeic, syringic, cinnamic, and gentistic acids, which has other health benefits and can have applications in the food, pharmaceutical, and cosmetic industries. In that case, according to the results obtained, the best technique for its recovery is PLE with ethanol. 

Flavonoids are somewhat better represented in the oil than in the root extracts, with certain compounds from the flavonols subgroup found in the highest amounts. For example, the highest quantity of rutin was detected in the oils recovered from compressed propane extraction at 313.15 K and 6 MPa. It is the highest registered quantity not only among the three seed oils tested, but also among all of the root extracts analyzed. However, the quantity of rutin in the seed oils is pronouncedly influenced by the type of extraction, operating conditions, and solvents applied—the lowest amount is registered in the oil recovered by scCO_2_ with ethanol as a co-solvent. The trends of the quercetin amount in the oils are in a complete analogy to those of rutin. Hence, in terms of oils rich in flavonols such as rutin, quercetin, myrecitin, and kaempferol-3-O-glycoside, recovery by compressed propane at 313.15 K and 6 MPa is definitely the right technique to apply. 

If, on the other hand, the target for the *A. lappa* root biomass transformation is a compound with a very high market price, then epicatechin gallate (representative of flavan-3-ol, which has a wide spectrum of health benefits) with a value of USD 210.00 for 0.5 × 10^−3^ kg should be targeted. In this case, the most efficient technique for its recovery from the roots will be PLE with water (26.274 ng/mg). 

The above data reveal the potential and perspectives of burdock biomass, as well as indicating some of the best-performing routes to recover either a single secondary metabolite or a group of bioactives from the biomass. Consequently, the burdock case study can be used as a generic example that demonstrates how to specify key technologies, involving green processes, and the appropriate operating conditions and solvents in order to transform any sustainable, abundant, underused, and neglected biomass into extracts rich in high value-added compounds with applications in a variety of industries targeting human health and wellbeing.

## 5. Willow

The willow genus (*Salix* spp.) is part of the Salicaceae family, which also includes poplars (*Populus* spp.). There are approximately 330–500 different willow species that grow primarily in the northern hemisphere. There is considerable diversity within the willow genus, which includes everything from tall trees to shrubs and small scrub plants [70].

### 5.1. Willow Extract’s Pharmacological Effects

Willow has been used as medicine since at least 1500 BCE, and ancient civilizations knew of the willow’s analgesic, antipyretic, and anti-inflammatory effects. After not being used very much throughout the Middle Ages, willow was rediscovered as a medicine in 1763, when the first more scientific studies of willow’s effect on fever and pain were carried out by Edward Stone. However, it was not until the 1820s that it was possible to isolate salicin from willow, which was the active substance. This led to the synthesis of salicylic acid in 1838 and then in 1897 acetylsalicylic acid in a pure and stable form. Acetylsalicylic acid was named aspirin in 1899 and has since been the most widely used and proven drug in the world.

Aspirin has become a very popular drug due to its wide range of uses, as in addition to acting as a pain reliever, fever reducer, and anti-inflammatory, in the right doses, it can also be a blood thinner, thus helping to lower the risk of blood clots. The success of aspirin has meant that it has become a model substance for the development of many new drugs with similar therapeutic properties (e.g., paracetamol). These substances go under the collective term NSAID (no-steroidal anti-inflammatory drugs). The problem with the increasing consumption of NSAIDs is the side effects that can occur when taken, and which typically affect the gastrointestinal system and can cause bleeding and perforation. It is therefore desirable to find alternatives that have the same positive effects as NSAIDs, but give rise to fewer side effects. Here, willow extract has proven to be of interest [71,72].

Experiments carried out with willow extracts show that the extracts have higher analgesic and anti-inflammatory effects than would be expected if only salicin were responsible for these effects. At the same time, the side effects associated with aspirin were considered reduced. This is due to the synergetic effect between the chemical compounds in the extracts [72,73,74]. Therefore, it is of high importance to extract all of the compounds from willow.

The main bioactive compounds in willow besides salicin and salicylic derivatives are phenolic compounds as phenolic acids and flavonoids. 

The composition of the bioactive substances varies both from willow species to willow species and from plant part to plant part [75,76,77]. Moreover, willow leaves, commonly discarded as waste after bark collection, represent valuable sources of bioactive compounds.

### 5.2. Willow Extract Concentration and Purification Steps 

As demonstrated, there is an unmet need of finding alternatives to NSAIDs while the anti-inflammatory effects of willow extracts are proven. It is thus not surprising that willow extracts are marketed as herbal medicines or as nutritional supplements in dry or aqueous form. Despite this, not many effective and sustainable extraction and processing methods are published covering the valorization of this high-value pharmaceutical source. 

The extraction procedure and solvent selection are a critical point, since they dictate the amount and type of compounds transferred to the extract. Solvent extractions, i.e., solid–liquid extraction, is commonly used for the isolation of bioactive compounds from plant material [78,79]. The recognized disadvantages of these conventional methods increased the interest in more selective and green extraction techniques. Although extraction in 80% methanol or acetone gives 3.3 and 2.2 higher phenolic acid yields, respectively, than when using pure water [80], extracts of willow are usually obtained using pure water extraction as acetone or methanol needs to be separated from the extract before it can be used. Limited information is available about the industrial production and properties of these extracts. Although water is considered a green solvent, large amounts of water are needed to extract a high yield of phenolic and flavonoid compounds from willow, making the process less sustainable if water is not reclaimed from the process. 

Ostolski et al. [81] introduced an eco-friendly supercritical CO_2_ extraction method for extracting phenolic compounds cheaply, safely, and with water as a co-solvent. In their study, the total phenolic and flavonoid concentration using scCO_2_, scCO_2_ with water as the co-solvent, and water in a solid–liquid extraction were compared. Generally, they observed the highest concentration of phenolic compounds after extraction using scCO_2_ with water as a cosolvent, followed by maceration in water. The lowest concentration was found for the extracts obtained with scCO_2_. The highest concentration of flavonoids was reported in the extracts obtained after extraction with ScCO_2_, due to the hydrophobic nature of this group of compounds.

Supercritical extraction and green solvents enable rapid mass transfer and increase penetration capacity into the sample matrix, resulting in quick and efficient extractions. However, as demonstrated by Ostolski et al. [81], scCO_2_ is not the best fluid to extract polar compounds such as phenolics. The addition of water to the extraction setup improved the effectiveness of the extraction of the polar phenolics. Further scCO_2_ extraction requires high pressures making the extraction process more energy intensive than water extraction.

Gligoric et al. [82] introduced the use of microwave-assisted extraction. MAE is acknowledged as a green, sustainable, and efficient extraction technique commonly used for the isolation of bioactive compounds with advantages that include a reduction in the extraction time, an enhanced extraction rate, and higher yields compared to conventional extraction methods. In their study, Gligoric et al. [82] applied MAE to six different willow species. They performed chemical characterization and examined the antioxidant capacity of bark and leaf extracts of these species. They found that the total phenolic amount in the bark and leaves varied. Phenolic acids were similarly distributed in the bark and leaves, while flavonoids were found in higher concentrations in the leaves than in the bark. When looking at the total phenolic content in the same species, higher amounts were observed in the bark than in the leaves. Unfortunately, from a processing point of view, they did not compare MAE with non-microwave-assisted water extraction, making it difficult to assess the advantage of MAE over traditional water extraction. In conclusion, at present, traditional water extraction with or without MAE seems the greener option.

Following the extraction step, the extract needs to be filtered to remove particulates and microorganisms, and in some cases, a form of sterilization of the product is needed before a final concentration step can be carried out to obtain a concentrate rich enough in phenolics and flavonoids to have a medical effect. Though little information on these steps is disclosed in the literature, the typical industrial procedure seems to be filtering through a 5 µm standard filter, followed by ultrafiltration to remove microorganisms and viruses, with the concentration being performed as vacuum evaporation to remove excess water. 

As vacuum evaporation is an energy-intensive process, Christensen et al. [83] introduced the use of reverse osmosis (RO) as the final step to concentrate the willow extract. The processing steps consisted of a 45 µm prefilter to remove larger solids from the raw willow extract, followed by microfiltration (MF, Alfa Laval FSMO 45PP, 0.45 µm) to remove microorganisms and smaller organic particulate residues, ultrafiltration (UF, Alfa Laval GR81PP, 10 kDa MWCO) to remove viral- and macromolecules that could foul the RO membrane, and finally the concentration was conducted using an RO membrane (Alfa Laval RO98pHt, 98% NaCl retention). Using this procedure, 99% recovery of dry matter in the original extract was possible, with the weight of dry matter increasing from 0.4 to 4%. The actual recovery of the phenolics and flavonoids could not be determined as their raw extract concentration was below the detection limit, but none was detected in the RO permeate. The RO permeate water corresponded to 55% of the original extraction water and can be reclaimed directly as extraction water for the willow extraction process, while 2% of the extract water ended up in the concentrate.

In special cases where the synergetic effects are not wanted, but a pure product is targeted, water extraction might be combined with chromatographic recovery and purification of the individual phenolics and flavonoids, as shown by Dou et al. [84]. They used a three-step chromatographic column separation setup to separate willow extract into purified phenolic and flavonoid compounds or fractions. They concluded that willow water extract should first be fractionated into a monosaccharide–colorant fraction, a picein–salicin-like compounds fraction, a pure triandrin fraction, and a pure (+)-catechin fraction using Sephadex G-10 as column material. The monosaccharide–colorant fraction can be separated further using CA10GC in its Na^+^ ion form as column material. This could be achieved using water as a solvent. The separation of picein and salicin-like compounds can be achieved using Amberchrom CG300M after triandrin has been separated from these compounds by Sephadex G-10. The recoveries from the Amberchrom CG300M column were 99.1% for salicin-like compounds and 97.5% for picein. For this separation, a solvent mixture of 10%-weight ethanol in water was necessary, making a separate ethanol recovery step necessary for the efficient use of this separation technique.

The conclusion is that a combination of water extraction, possibly using MAE, with RO concentration would be a promising energy and water-efficient processing method. This could be combined with chromatographic separation either after the UF or the RO step, should a purified product be of interest.

It is also apparent that further experimental work is needed to be able to determine the optimal combination of these processes both with respect to energy consumption and water/ethanol recycling.

## 6. Conclusions

The studies presented in this review demonstrate that converting biowastes/biomass into high-value products represents a valuable opportunity for the overall economy. 

Diverse compounds can be obtained from the considered biomasses, with uses in different applications. BSG is a source of proteins, lipids, waxes, gums, resins, tannins, and essential oils. The primary focus of extracting the lipid fraction from SCG is due to the presence of various beneficial compounds, including triacylglycerols, diacylglycerols, diterpene alcohol esters, fatty acids, sterols, caffeine, cafestol, and kahweol. These compounds have been extensively studied for their potential health benefits, such as anticarcinogenic and antioxidant effects. 

The use of the lipid fraction from spent coffee grounds, particularly the triacylglycerols, from which it is possible to obtain biodiesel through transesterification, is already a practice in a few countries due to the lack of other usual matrices to produce vegetable oils. On the other hand, the diterpenes identified, such as cafestol and kahweol, have a serum lipid-raising effect, while showing multiple potential pharmacological actions such as anti-inflammatory, hepatoprotective, anti-cancer, anti-diabetic and anti-osteoclastogenic activities.

Bioactive lignans, and a rich variety of secondary metabolites and high-value antioxidants such as linoleic, chlorogenic, cinnamic, caffeic, *o*-coumaric, syringic, gentisitic, etc. acids, as well as sugars, can be recovered from burdock (*Arctium lappa*). It was evidenced that the chemical composition and metabolite content of the plant’s extracts depend on the technique and operating conditions applied, and can vary due to genotype, seasonality, and cultivation conditions.

In the case of willow, phenolic compounds such as phenolic acids and flavonoids are the main bioactive substances, alongside salicin and salicylic derivatives. These compounds vary not only between different species of willow, but also among different parts of the same plant. Willow leaves, which are typically thrown away after the bark is collected, have been found to contain valuable bioactive compounds. 

All of the data presented highlights that the utilization of biomass needs to be achieved through sustainable extraction methods, such as microwave-assisted extraction, ultrasound-assisted extraction, and extraction with compressed fluids applying GRAS co-solvents, while also considering recycling of the extraction media. This aligns with the principles of the “green” economy, and the implementation of the European Green Deal provided that the entire supply chain is analyzed from a sustainability point of view. 

In conclusion, the consideration reported in the introduction regarding the importance of extraction techniques in processing biomass seems to be supported by the cases considered. However, the variability of the biomass is still a critical point, and efforts are required toward making the processes adaptable to the change in feed characteristics. This is a challenge that tools such as artificial intelligence and innovative sensors can help to resolve to make industrial production sustainable and competitive. Furthermore, familiarity with the design and optimization procedures applied to conventional and alternative extraction processes are expected to gain a spotlight in the education of new chemical and biochemical engineers. 

## Figures and Tables

**Table 1:** BSG chemical composition based on different sources.

	Reference				
Component (% DW)	[17]	[18]	[19]	[20]	[21]
Cellulose	16.8	nd	25.58 ± 6.27	nd	21.47 ± 1.22
Hemicellulose	28.4	nd	21.51 ± 0.73	nd	30.95 ± 1.55
- Xylan	nd	nd	12.47 ± 1.30	nd	nd
- Arabinan	nd	nd	5.85 ± 0.82	nd	nd
Lignin	27.8	23.39 ± 0.56	12.72 ± 2.00	12.0 ^b^	6.94 ± 0.95
Carbohydrate	nd	34 ^a^	nd	46.2 ± 0.06 ^a^	nd
Starch	nd	1.48 ± 0.01	nd	nd	nd
Proteins	15.2	23.10 ± 0.09	31.81 ± 0.46	26.9 ± 0.1	23.07 ± 0.13
Lipids	nd	13.51 ± 0.78	nd	11.5 ± 0.03	8.09 ± 0.17
Acetyl groups	1.4	nd	nd	nd	nd
Extractives	5.8 ^a^	nd	nd	nd	nd
Polyphenols	nd	1.70 ± 0.02	nd		nd
Ashes	4.6	3.29 ± 0.06	3.07 ± 0.03	3.40 ± 0.04	3.86 ± 0.03

^a^ value estimated by difference; ^b^ value estimated as 12% *w*/*w*; nd – not detected.

## Data Availability

Not applicable.
